# Detection of PCV2d in pig industry of the Iberian Peninsula

**DOI:** 10.1007/s11259-025-10874-x

**Published:** 2025-09-03

**Authors:** Sara Gomes-Gonçalves, Sérgio Santos-Silva, Guilherme Moreira, Andreia V. S. Cruz, João R. Mesquita

**Affiliations:** 1 School of Medicine and Biomedical Sciences (ICBAS), Universidade do Porto (UP), Porto, 4050-313 Portugal; 2 Associate Laboratory for Animal and Veterinary Science (AL4AnimalS), Lisboa, 1300-477 Portugal; 3 Centro de Estudos de Ciência Animal (CECA), Instituto de Ciências, Tecnologias e Agroambiente (ICETA), Universidade do Porto (UP), Porto, 4051-401 Portugal

**Keywords:** Porcine circovirus type 2d, Detection, PCR, Domestic pig

## Abstract

Porcine circovirus type 2 (PCV2) is a major viral pathogen affecting swine worldwide, causing significant economic losses through its association with porcine circovirus-associated diseases (PCVD), which can lead to reproductive failure, growth retardation, and increased mortality in affected herds. Although vaccination against the PCV2a and PCV2b genotypes has reduced clinical disease, the PCV2d genotype is increasingly prevalent in many regions. Molecular detection was performed on fecal samples from collected from a slaughterhouse using PCR targeting the ORF2 gene to detect PCV2 genotypes. Of 400 samples tested, 8.5% (17/200; 95% CI: 5.03–13.26) of samples from Portugal were positive for PCV2d, while none from Spain tested positive. This study reports the first confirmed detection of PCV2d in domestic pigs in Portugal in indexed journals, indicating circulation within the Portuguese swine population. The absence of PCV2 in Spanish samples may reflect differences in regional epidemiology or biosecurity practices. These findings highlight the importance of ongoing molecular surveillance and farm-level monitoring to inform vaccination strategies and control measures in response to evolving PCV2 genotypes. Integrated approaches should also consider wildlife reservoirs and environmental factors to improve management of PCV2 spread in domestic swine.

## Introduction

Swine infectious diseases profoundly impact the livestock breeding industry, international trade, and human public health (Guo et al. 2022). Porcine circovirus type 2 (PCV2) is a small, non-enveloped, single-stranded DNA virus within the family *Circoviridae* noted for having one of the highest mutation rates among DNA viruses (Gillespie et al. 2009; Correa-Fiz et al. 2020). It is the primary etiological agent of porcine circovirus-associated diseases (PCVAD), a group of clinical syndromes that include systemic disease, reproductive failure, enteritis, and subclinical infections (Gillespie et al. 2009; Shi et al. 2021). Additionally, PCV2 is one of the primary pathogens involved in Porcine Respiratory Disease Complex (PRDC), where it contributes to respiratory illnesses by causing histiocytic and granulomatous interstitial lung lesions, as well as proliferative and necrotizing pneumonia, often in combination with other viral infections (D’Annunzio et al. 2023). This respiratory condition is referred to as PCV2-associated respiratory disease or PCV2-lung disease (Opriessnig et al. 2007). Another manifestation of PCV2 infection is reproductive failure in breeding herds, characterized mainly by an increased number of mummified and non-viable piglets at birth (Madson and Opriessnig 2011). PCV2 infection compromises immune function in pigs, increasing susceptibility to secondary infections and reducing the effectiveness of vaccine-induced immunity (Guo et al. 2022). These conditions exert a considerable burden on global pig production, as a threat to pig industry since its discovery in the early 90’s, with PCVAD-associated losses mortality ranging from 4 to 20% and economic losses estimated to exceed €500 million annually in Europe alone (Correa-Fiz et al. 2020; MSD Animal Health 2025).

Despite the widespread deployment of PCV2a or PCV2b-based vaccines, which have reduced clinical disease, PCV2 remains highly prevalent (Karuppannan and Opriessnig 2017). Since the early 2010 s, there has been a notable global shift from PCV2b to PCV2d, a genotype that is now dominant in many swine-producing regions (Karuppannan and Opriessnig 2017). PCV2d has been associated with higher viremia, increased transmission efficiency (Kim and Hahn 2021). Current PCV2 vaccines are considered “leaky vaccines”, as they do not completely eliminate viral replication or transmission as a result, the virus continues to circulate even in vaccinated herds, which may contribute to viral persistence and genotype shifts due to ongoing immune selection pressure (Opriessnig et al. 2013; Seo et al. 2014; Li et al. 2025).

The Iberian Peninsula is formed by Spain and Portugal, and was recently considered the most intensive pig-producing regions in Europe in 2024 with pig slaughter volumes exceeding 50,000 and 5,000 heads per thousand pigs, in Spain and Portugal’s production systems, respectively (European Commission Directorate-General for Agriculture and Rural Development 2025). However, molecular surveillance of PCV2 genotypes in Portugal remains sparse. While Spain has documented the rise of PCV2d, no prior studies have confirmed its presence in domestic pigs in Portugal (Sibila et al. 2021). PCV2d was previously detected in wild boar in Portugal (de Sousa Moreira et al. 2022), but its circulation in farmed pigs has not yet been reported.

Given the fecal-oral route of PCV2 transmission, stool sampling at slaughterhouses provides a practical and non-invasive method for detecting subclinical infections and assessing herd-level exposure (Yang et al. 2003). Such surveillance is especially valuable in detecting emerging genotypes that may not yet be associated with overt disease.

This study aimed to investigate the potential circulation of PCV2 in the Iberian Peninsula pigs by conducting molecular screening of fecal samples collected from a commercial slaughterhouse. Our findings represent the detection of PCV2d in domestic pigs in Portugal. This evidence of viral circulation suggests distinct epidemiological patterns between Portugal and neighboring Spain and raises concerns about potential gaps in biosecurity or monitoring systems. These results emphasize the need to expand molecular surveillance and reassess control strategies in light of the evolving PCV2 genotype landscape.

## Materials and methods

### Sampling

A total of 400 fecal samples were collected from a commercial slaughterhouse located in the northern Portuguese city of Porto carried out over a two-month period, specifically in December 2021 and January 2022. The selection of this site was based on logistical feasibility and aimed to provide a preliminary overview of PCV2 circulation. The samples were retrieved directly from the small intestine in the visceral processing area prior to intestinal cleaning of non-diarrheic fattened pigs, of five swine farms from both Spain and Portugal. A total of 200 samples were obtained from animals raised on two farms in northern Spain near Santiago de Compostela and the remaining 200 from pigs raised on three farms in northern Portugal near the Porto region. Importantly, no animals were sacrificed specifically for this study. Samples were maintained at 4 °C during transport and delivered to the laboratory within 12 h of collection. Upon arrival, all samples were stored at − 80 °C until nucleic acid extraction was conducted. This same sampling was previously used in (Santos-Silva et al. 2025).

### DNA extraction

DNA extraction was carried out by first diluting the stool samples to a 10% concentration using phosphate-buffered saline (PBS, pH 7.2), with 500 µL of PBS added to each sample. The suspensions were vortexed thoroughly to ensure homogenization, then centrifuged at 8,000×g for 5 min (Eppendorf, Hamburg, Germany). From the resulting supernatant, 140 µL was used for DNA extraction and purification using the QIAamp DNA Mini Kit (Qiagen, Hilden, Germany), following the manufacturer’s guidelines. The extraction process was automated on the QIAcube^®^ platform (Qiagen). Purified DNA was eluted in RNase-free water and stored at − 80 °C until further analysis.

### Molecular detection of PCV2

The molecular detection of PCV2 was performed using conventional polymerase chain reaction (PCR) targeting the open reading frame 2 (ORF2), which encodes the capsid protein. Amplification was carried out using the primer pair PCV2all_F and PCV2all_R, designed to yield a 685 bp amplicon, as previously described (Oliver-Ferrando et al. 2016). Each PCR reaction was set up in a final volume of 25 µL using the NZYTaq II 2× Green Master Mix (NZYTech, Lisbon, Portugal), in accordance with the manufacturer’s protocol. Reactions were run on a Bio-Rad thermocycler (Bio-Rad, Hercules, CA, USA).

Both positive control (DNA from a previously confirmed PCV2-positive sample (de Sousa Moreira et al. 2022) and a no-template control (reaction mixture with RNase-free water replacing the DNA template) were included in each PCR run to ensure validity and exclude contamination. The thermal cycling protocol consisted of an initial denaturation at 95 °C for 3 min, followed by 40 amplification cycles comprising 94 °C for 30 s, 53 °C for 30 s and 72 °C for 15 s, with a final extension step at 72 °C for 10 min.

PCR products were analyzed by electrophoresis on 1.5% agarose gels stained with Xpert Green Safe DNA gel dye (GRiSP^®^, Porto, Portugal). Electrophoresis was conducted at a constant voltage of 120 V for 25 min. Amplicons were visualized under ultraviolet light to confirm successful amplification.

### Sequencing and phylogenetic analysis

Amplicons of the expected size were purified using the GRS PCR and Gel Band Purification Kit (GRiSP^®^, Porto, Portugal) and sequenced bidirectionally via Sanger sequencing. The resulting sequences were edited, aligned, and analyzed using the BioEdit Sequence Alignment Editor (v7.2.5). Consensus sequences were compared to the NCBI GenBank database using BLASTn (NCBI 2025).

Phylogenetic analysis was performed using 38 full-length sequences retrieved from the GenBank database. Sequence alignment was carried out with MAFFT v7.490, employing the L-INS-i algorithm for enhanced accuracy with diverse sequences. A Maximum Likelihood phylogenetic tree was constructed using IQ-TREE, with 1000 bootstrap replicates and the TIM3 + F + I + R2 as best substitution model. PCV1 was used as the outgroup lineage due to its evolutionary proximity. Additional annotations and visual enhancements were produced using the Interactive Tree of Life (iTOL) platform v7.

### Statistical analysis

The occurrence of PCV2. in domestic pigs was calculated as the proportion of positive samples relative to the total number analyzed, with a 95% confidence interval (95% CI). Data processing and preliminary analyses were performed using Microsoft Excel^®^ for Microsoft 365 MSO (Version 2312, Build 16.0.17126.20132, 64-bit).

## Results

Out of a total of 400 swine stool samples collected across the Iberian Peninsula, only samples from Portugal tested positive for PCV2, specifically the PCV2 genotype d. The observed prevalence in Portugal was 8.5% (17/200; 95% CI: 5.03–13.26), while no PCV2 was detected in Spanish swine represented in Table 1. This difference in occurrence between Portugal and Spain was statistically significant (Fisher’s Exact Test, *p* < 0.001).

**Table 1:** Prevalence of PCV2d in swine samples from Iberian Peninsula

Country	Nº of samples	Nº of Positives	Genotype	% Positives	Test Result (Fisher *p*-value)
Portugal	200	17	PCV2d	8.5	*p* < 0.001
Spain	200	-	-	0	

**Fig. 1:**
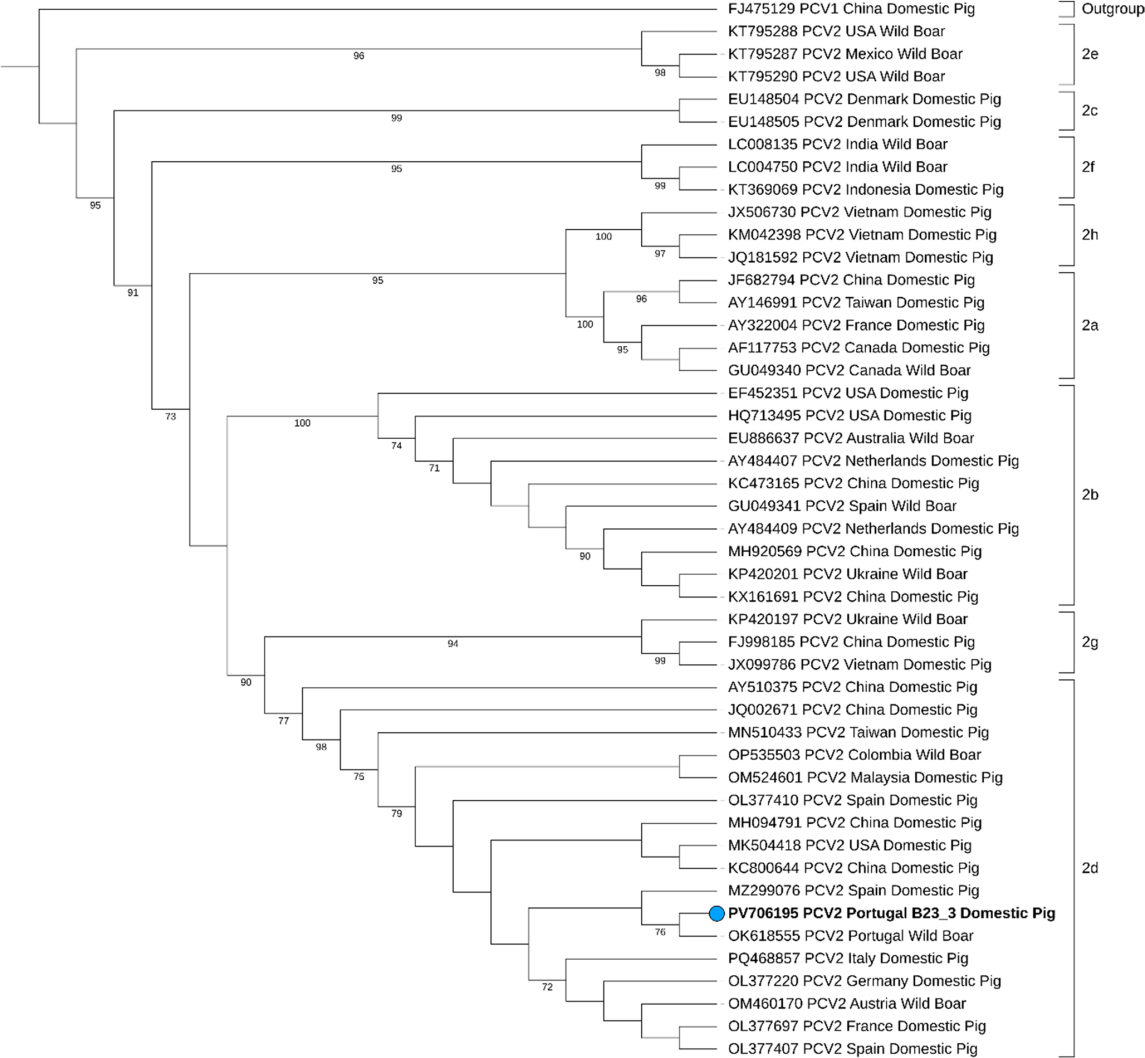
The phylogenetic tree of PCV2 genotypes was constructed using IQ-TREE software, based on ORF2 gene (capsid protein) reference sequences from the GenBank database. The representative sequence generated in this study is indicated by a blue circle and bold formatting. Reference sequences retrieved from GenBank are shown without formatting, along with their respective accession numbers and geographic location. The tree was inferred using the TIM3 + F + I + R2 substitution model, with 1000 bootstrap replicates to ensure robustness. Bootstrap values of 70% or higher are displayed in the figure. Additional annotations and visual enhancements were produced using the Interactive Tree of Life (iTOL) platform v7

All sequences obtained through BLASTn analysis (NCBI 2025) showed 99.84% nucleotide identity with previous reference PCV2 strains. Every sequence obtained in this study had a single nucleotide polymorphism (SNP) at position 498 of the ORF2 gene (G → A). The highest similarity hits are shown in Table 2. Since all sequences obtained were genetically identical with each other, the one with the greatest) length (isolate B23_3 PV706195 was selected as the reference for further analysis. Phylogenetic analysis based on the ORF2 gene, which encodes the capsid protein, was conducted to confirm the genotype, as shown in Fig. 1.

**Table 2:** Highest matches obtained by BLASTn analysis

Accession nº	Identity (%)	Host	Location
OM524601	99.84	Domestic pig	Malaysia
PQ468857	99.84	Domestic pig	Italy
OP535503	99.84	Wild boar	Colombia
OL377220	99.84	Domestic pig	Germany
OL377697	99.84	Domestic pig	France
OM460170	99.84	Wild boar	Austria
OK618555	99.84	Wild boar	Portugal

The sequences obtained in this study were deposited on GenBank with accession PV706195-PV706211.

## Discussion

This study provides to the best of our knowledge the first confirmed detection of the PCV2d genotype in domestic pigs in Portugal in indexed journals, suggesting widespread dissemination of this genotype in the Iberian Peninsula. Until now, PCV2d circulation in Portugal had only been documented in wild boar, with no prior reports in farmed swine (de Sousa Moreira et al. 2022). To the best of our knowledge the last molecular study on the circulation of PCV2 in Portugal, performed in 2011, reported the presence of PCV2a and PCV2b, genotypes but did not detect PCV2d (Henriques et al. 2011). Although information on the presence of PCVD in local nursery pigs is unavailable, in Portugal, pigs can only be slaughtered if they pass an ante mortem veterinary inspection confirming they are clinically healthy (Governo de Portugal 1994). The detection of PCV2d in 8.5% of fecal samples from such animals at commercial slaughterhouses therefore indicates subclinical viral circulation and suggests possible gaps in current biosecurity or surveillance practices. The presence of PCV2d in asymptomatic animals underscores the subclinical nature of many PCV2 infections and suggests that the virus may be spreading undetected within farms (Chen et al. 2024). Slaughterhouse sampling, while not a substitute for farm-level epidemiological surveillance, offers a practical proxy for assessing broader regional circulation. Given the fecal–oral transmission of PCV2, positive stool samples likely reflect recent or ongoing exposure and viral shedding at the herd level (Yang et al. 2003). The fact that no PCV2 was detected in Spanish samples collected under the same conditions is noteworthy and may reflect real differences in viral dynamics or production practices at the country level. One possible explanation is that more effective vaccination strategies in Spain may have reduced viral shedding. However, these findings should be interpreted with caution. The sample size may not have been sufficient to detect low-prevalence infections, especially considering that PCV2d has previously been reported in Spain (Sibila et al. 2021). Additionally, the absence of a physical barrier between Portugal and Spain, along with the potential role of wild boar in PCV2 transmission, suggests that cross-border viral movement remains a possible contributing factor. These considerations indicate that further investigation would be useful to clarify the underlying causes. Despite data was not available regarding the vaccination status on these farms, important considerations regarding vaccination strategies should be made. Although the available PCV2a- and PCV2b-based vaccines have demonstrated adequate cross-protection in controlled experimental settings, even against PCV2d (Karuppannan and Opriessnig 2017). Field evidence from other regions has associated PCV2d outbreaks with incomplete protection in vaccinated herds (Li et al. 2025). This apparent discrepancy reinforces the characterization of current vaccines as “leaky”, they significantly reduce clinical signs and improve productivity but do not prevent viral replication or transmission (Bandrick et al. 2022; Chen et al. 2023). Such partial immunity may sustain viral circulation and contribute to the emergence of fitter genotypes through immune selection pressure. In this context, the rising prevalence of PCV2d globally, and now its detection in Portugal, may reflect the evolutionary advantage of genotypes capable of evading vaccine-mediated immune systems.

The presence of PCV2d has been previously described in Europe countries namely Italy, Belgium and Spain, scoring the shift of genotypes withing PCV2 (Wei et al. 2019; Sibila et al. 2021; Chen et al. 2023; Faustini et al. 2024). The prior identification of PCV2d in wild boar in Portugal and its genetic proximity with the isolates of this study adds another dimension to the surveillance narrative (de Sousa Moreira et al. 2022). Wild boards can act as reservoirs for diverse swine important pathogens including PCV2 (Nisavic et al. 2022). The risk of transmission between wild and domestic populations increases, particularly in semi-intensive and free-range farming systems where livestock and wild fauna share habitats (Fanelli et al. 2022). This observation calls for a broader One Health approach that integrates surveillance across domestic animals, wildlife and the environment. The presence of PCV2 in environmental matrices such as surface water, manure, and shared grazing areas may serve as sentinel indicators of wider PCV2 circulation and evolutionary dynamics. responses (Zhai et al. 2019; Fanelli et al. 2022). The evolutionary capacity of PCV2 is well-documented (Franzo et al. 2016). Despite being a DNA virus, it exhibits mutation and recombination rates comparable to RNA viruses (Wen and He 2021). This genetic plasticity facilitates the rapid emergence and displacement of genotypes in response to ecological and immunological pressures.

While this study provides essential baseline data, several limitations must be acknowledged. The cross-sectional design prevents assessment of temporal trends or longitudinal infection dynamics. The lack of clinical correlation limits inferences regarding virulence or production impact. Publicly available data on breeding density, farm structure, and vaccine implementation protocols in both Portugal and Spain are limited or inconsistent, which constrained the ability to incorporate quantitative background comparisons. Furthermore, full genome sequencing was not performed, restricting insight into potential recombination events or antigenic drift within the PCV2d lineage. To address these gaps, subsequent research should prioritize expanded sample size, longitudinal sampling, genomic analysis and serological studies at the farm level to contextualize viral presence and evaluate the effectiveness of current immunization strategies. It will also be important to investigate related variables in greater detail, with particular emphasis on vaccination coverage, biosecurity implementation, detection method sensitivity and inclusion of additional serological testing data. In summary, the detection of PCV2d in Portugal’s domestic swine herd represents both a novel finding and a call for enhanced molecular surveillance. As PCV2 continues to evolve and adapt, coordinated regional efforts are essential to ensure timely detection, effective immunization, and sustainable control of PCVAD in European pig production.

## Conclusion

This study provides the first documented evidence of PCV2d circulation in domestic pigs in Portugal in indexed journals, marking an important shift in the regional epidemiology of porcine circovirus. The detection of this genotype in asymptomatic animals at slaughterhouses underscores its subclinical nature and reveals potential gaps in biosecurity and molecular surveillance. The absence of PCV2 detection in Spanish samples collected under the same protocol highlights either genuine differences in prevalence or limitations in sampling scope, and it emphasizes the need for broader geographic monitoring.

Continued molecular surveillance at the farm level, complemented by environmental and wildlife monitoring, will be essential to anticipate genotype shifts, refine vaccine strategies, and protect swine health and productivity in Portugal and beyond.

## Data Availability

No datasets were generated or analysed during the current study.
